# The Impact of Influenza and Tuberculosis Interaction on Mortality Among Individuals Aged ≥15 Years Hospitalized With Severe Respiratory Illness in South Africa, 2010–2016

**DOI:** 10.1093/ofid/ofz020

**Published:** 2019-03-19

**Authors:** Sibongile Walaza, Stefano Tempia, Halima Dawood, Ebrahim Variava, Nicole Wolter, Andries Dreyer, Jocelyn Moyes, Claire Von Mollendorf, Meredith McMorrow, Anne Von Gottberg, Sumayya Haffejee, Marietje Venter, Florette K Treurnicht, Orienka Hellferscee, Neil A Martinson, Nazir Ismail, Cheryl Cohen

**Affiliations:** 1 Centre for Respiratory Diseases and Meningitis, National Institute for Communicable Diseases of the National Health Laboratory Service, Johannesburg, South Africa; 2 School of Public Health, Faculty of Health Sciences, University of the Witwatersrand, Johannesburg, South Africa; 3 Influenza Division, Centers for Disease Control and Prevention, Atlanta, Georgia; 4 Influenza Program, Centers for Disease Control and Prevention, Pretoria, South Africa; 5 Pietermaritzburg Metropolitan Hospital Complex, KwaZulu-Natal, South Africa; 6 Department of Medicine, Klerksdorp Tshepong Hospital, North West Province; 7 School of Clinical Medicine, Faculty of Health Sciences, University of Witwatersrand, Johannesburg, South Africa; 8 Perinatal HIV Research Unit, MRC Soweto Matlosana Collaborating Centre for HIV/AIDS and TB; 9 School of Pathology, Faculty of Health Sciences, University of the Witwatersrand, Johannesburg, South Africa; 10 Centre for Tuberculosis, National Institute for Communicable Diseases of the National Health Laboratory Service, Johannesburg, South Africa; 11 Division of Global Health Protection, Centers for Disease Control and Prevention, Pretoria, South Africa; 12 DST/NRF Centre of Excellence for Biomedical Tuberculosis Research, University of the Witwatersrand, Johannesburg, South Africa; 13 Johns Hopkins University Center for TB Research, Baltimore, Maryland; 14 Faculty of Health Sciences, University of Pretoria, Pretoria; 15 Zoonosis Research Program, Department of Medical Virology, University of Pretoria, Pretoria, South Africa

**Keywords:** coinfection, HIV, influenza, mortality, South Africa, tuberculosis

## Abstract

**Background:**

Data on the prevalence and impact of influenza–tuberculosis coinfection on clinical outcomes from high–HIV and –tuberculosis burden settings are limited. We explored the impact of influenza and tuberculosis coinfection on mortality among hospitalized adults with lower respiratory tract infection (LRTI).

**Methods:**

We enrolled patients aged ≥15 years admitted with physician-diagnosed LRTI or suspected tuberculosis at 2 hospitals in South Africa from 2010 to 2016. Combined nasopharyngeal and oropharyngeal swabs were tested for influenza and 8 other respiratory viruses. Tuberculosis testing of sputum included smear microscopy, culture, and/or Xpert MTB/Rif.

**Results:**

Among 6228 enrolled individuals, 4253 (68%) were tested for both influenza and tuberculosis. Of these, the detection rate was 6% (239/4253) for influenza, 26% (1092/4253) for tuberculosis, and 77% (3113/4053) for HIV. One percent (42/4253) tested positive for both influenza and tuberculosis. On multivariable analysis, among tuberculosis-positive patients, factors independently associated with death were age group ≥65 years compared with 15–24 years (adjusted odds ratio [aOR], 3.6; 95% confidence interval [CI], 1.2–11.0) and influenza coinfection (aOR, 2.3; 95% CI, 1.02–5.2). Among influenza-positive patients, laboratory-confirmed tuberculosis was associated with an increased risk of death (aOR, 4.5; 95% CI, 1.5–13.3). Coinfection with other respiratory viruses was not associated with increased mortality in patients positive for tuberculosis (OR, 0.7; 95% CI, 0.4–1.1) or influenza (OR, 1.6; 95% CI, 0.4–5.6).

**Conclusions:**

Tuberculosis coinfection is associated with increased mortality in individuals with influenza, and influenza coinfection is associated with increased mortality in individuals with tuberculosis. These data may inform prioritization of influenza vaccines or antivirals for tuberculosis patients and inform tuberculosis testing guidelines for patients with influenza.

Both influenza and tuberculosis disease cause significant morbidity and mortality globally [1–4]. Data on the impact of influenza and tuberculosis coinfection (tuberculosis coinfection refers to tuberculosis disease), particularly from high–HIV and –tuberculosis burden settings are limited. Most available data are from ecologic or individual-level studies, usually conducted during pandemic influenza years, frequently from descriptive studies, with limited sample size and lacking information on important confounders such as HIV infection [5–11]. A better understanding of influenza and tuberculosis interaction is important to assist policy makers to make decisions regarding priority target groups for influenza immunization.

HIV is associated with increased risk of tuberculosis and influenza-associated morbidity and mortality [12–15]. In South Africa, 11.6%–12.7% of the general population and approximately one-fifth (22.2%) of reproductive-age women were living with HIV between 2010 and 2016 [16]. In 2015, there were an estimated 450 000 incident cases of tuberculosis, of whom 57% were HIV infected. There were an estimated 25 000 and 73 000 deaths among HIV-negative and HIV-positive tuberculosis patients, respectively, in 2015 [17]. Similarly, an increased risk of influenza-associated hospitalization and mortality has been reported among individuals with AIDS [13, 14, 18, 19]. We previously reported an increased risk of mortality in patients with influenza–tuberculosis coinfection from 6 hospitals in South Africa [20]. However, this earlier study was limited by differential enrollment criteria across different participating sites and a sample size that did not allow us to evaluate specific interactions in the influenza- and tuberculosis-infected groups separately.

In this study, we assess the effect of influenza–tuberculosis coinfection on mortality among patients hospitalized with severe respiratory illness, first among those who were tuberculosis positive and second among those that were influenza positive.

## METHODS

### Study Design

We conducted prospective hospital-based sentinel surveillance for severe respiratory illness (SRI) at 2 hospitals in 2 provinces, Edendale Hospital in the KwaZulu-Natal Province and Tshepong Hospital in the North West Province, from June 2010 through May 2016.

### Study Inclusion

A case of SRI was defined as admission with physician-diagnosed lower respiratory tract infection (LRTI), including suspected tuberculosis of any duration. Patients aged ≥15 years from the hospital catchment area were included in our analysis.

### Study Procedures

SRI cases admitted from 17H00 on Sunday through 13H00 on Friday were eligible for enrollment. Data on clinical presentation, medical history, antiretroviral therapy for HIV-infected individuals, inpatient investigations, clinical management, and outcome were collected through structured interviews and record review. Each patient was followed up until discharge. Treatment decisions and diagnostic tests for patient management were determined by the attending physician.

### Sample Collection and Laboratory Procedures

Combined nasopharyngeal (NP) and oropharyngeal (OP) swabs and blood specimens were systematically collected from consenting patients by study staff. Combined NP and OP swabs were transported in universal transport medium (Copan, Brescia, Italy) on cooled ice packs to the National Institute for Communicable Diseases (NICD) within 72 hours of collection and were tested for influenza A and B viruses, respiratory syncytial virus, parainfluenza virus types 1, 2, and 3, adenoviruses, human metapneumovirus, enterovirus and rhinoviruses, using either a multiplex real-time reverse transcription polymerase chain reaction (PCR) assay [21] from 2010 through to 2014 or the commercial Allplex Respiratory Assay (panels 2 and 3; Seegene, Seoul, Korea) from 2015. Influenza A and B and respiratory syncytial viruses were tested using the commercial FTD Flu/RSV assay (Fast Track Diagnostics, Luxembourg) from 2015 to 2016. Parainfluenza virus types 1, 2 and 3, adenoviruses, human metapneumovirus, enteroviruses, and rhinoviruses were only tested for the period 2010–2015.

From June 2010 to May 2012, tuberculosis testing of expectorated sputum was conducted at the discretion of the attending clinician. Systematic testing for tuberculosis was instituted in June 2012. For this period, for enrolled patients who were not tested for tuberculosis as part of clinical management, expectorated sputum (or induced sputum if the patient was not able to expectorate) was collected by study staff for tuberculosis testing at the hospital laboratory, and a second sample was collected and sent to NICD in Johannesburg for tuberculosis and other bacterial pathogen testing. Sputum samples were frozen at –20°C and stored at the site laboratory until shipped on dry ice (on a weekly basis) to NICD. Microbiological investigation for tuberculosis at the site laboratory was performed by smear microscopy, culture, and/or XpertMTB/Rif (Cepheid, Sunnyvale, CA). At NICD, tuberculosis was identified by microscopy and culture only. Smear microscopy of sputum samples was performed using fluorescent auramine-O staining for acid-fast bacilli (AFB). Culture was performed at the site laboratory and at NICD using liquid media with the Bactec MGIT 960 (Becton Dickinson, NJ) system. Positive cultures were identified as *Mycobacterium tuberculosis* complex using Ziehl-Neelsen staining and MPT64 antigen testing (Becton Dickinson, NJ) [22]. Patients without an available sputum for testing were considered not tested for tuberculosis and were excluded from the models. Among patients tested for tuberculosis, a patient was considered tuberculosis positive if at least 1 tuberculosis test (smear, culture, or PCR) was positive. Whole-blood specimens were tested for the presence of pneumococcal DNA (*lyt*A gene) using quantitative real-time PCR [23].

### Determination of HIV Status and CD4 Count Testing for HIV-Infected Patients

HIV status was obtained from several data sources. HIV testing was requested by admitting physicians as part of clinical management for most patients. This included a rapid HIV test with confirmation by a second HIV rapid test if the first sample was positive. Based on National guidelines for HIV testing, discrepant results from the 2 rapid tests were confirmed by an HIV enzyme-linked immunosorbent assay (ELISA) [24]. For consenting patients who were not tested by the attending clinician, pretest counseling and bedside testing for HIV were offered by NICD study staff or an HIV ELISA test was performed at NICD using a dried blood spot or whole-blood specimen [25]. Homemed’s HIV1/2 point of care test (POCT) was used as the first screening test, and the ABON HIV1/2 Tri-Line test kit was used as the second confirmatory test for all participants testing positive on the first screening test. When results were available from multiple sources or were discrepant, the NICD result was used. CD4+ cell count per cubic millimeter was collected for HIV-infected patients as part of clinical management or part of the study if not requested by the clinician.

### Ethical Consideration

The protocol was approved by the University of the Witwatersrand Human Research Ethics Committee (reference Nos. M081042 and M1400824) and the University of KwaZulu-Natal Human Biomedical Research Ethics Committee (Nos. BF157/08 and BE 496/14).

### Analysis

We implemented multivariable logistic models to assess the association between tuberculosis and influenza coinfection on mortality in 2 different populations:

- all individuals testing tuberculosis positive (who were also tested for influenza);
- all individuals testing influenza positive (who were also tested for tuberculosis).

Factors associated with testing for tuberculosis were assessed for potential bias in the population that received tuberculosis testing. Symptom duration, HIV serostatus, and age were included a priori in models (a) and (b) as important potential confounders. Symptom duration was included, based on our previous findings demonstrating that influenza–tuberculosis coinfection was associated with increased risk of death compared with tuberculosis or influenza single infection in patients with symptoms >7 days, but not in patients with acute presentation [20].

Factors significant at *P* < .1 on univariate analysis were evaluated in the multivariable analysis, and nonsignificant factors at *P* ≥ .05 were dropped from the multivariable model using stepwise forward selection. All 2-way interactions in the final multivariable additive model were evaluated. Two-sided *P* values <.05 were considered significant throughout. Stata, version 14 (StataCorp Limited, College Station, TX), was used for the analysis.

## RESULTS

From June 2010 through May 2016, we enrolled 6228 individuals aged ≥15 years with SRI. Of enrolled patients, 6149 (99%) and 4300 (69%) were tested for influenza and tuberculosis, respectively. The overall HIV prevalence among 5853 patients aged ≥15 years with SRI and known HIV results was 76% (4445/5853), 69% (213/308) among influenza-positive cases, and 83% (869/1043) among tuberculosis-positive cases. Overall mortality among enrolled patients was 13% (777/6064); it was 11% (119/1088) among tuberculosis-positive cases, 12% (37/318) among influenza-positive cases, and 19% (8/42) among influenza–tuberculosis-coinfected cases.

Compared with patients not tested for tuberculosis, on multivariable analysis, patients tested for tuberculosis were more likely to present with cough (adjusted odds ratio [aOR], 2.1; 95% confidence interval [CI], 1.5–2.8) and night sweats (aOR, 1.3; 95% CI, 1.1–1.6), to have a history of working in a mine (aOR, 1.6; 95% CI, 1.2–2.2), to have pneumococcal coinfection identified by *lyt A* PCR (aOR, 1.3; 95% CI, 1.0–1.8) and to be admitted at Tshepong Hospital (aOR, 1.5; 95% CI, 1.2–1.8); however, they were less likely to be admitted for >7 days compared with <3 days (aOR, 0.8; 95% CI, 0.6–0.9) and to die during admission (aOR, 0.5; 95% CI, 0.4–0.6) (Table1).

**Table 1.** Demographic and Clinical Characteristics of Individuals Aged ≥15 Years Admitted With Severe Respiratory Illness, by Tuberculosis Testing Status, at Tshepong and Edendale Hospitals, South Africa, June 2010–May 2016

Characteristic		Tested for Tuberculosis, n/N (%)	Unadjusted Odds Ratio (95% CI)	*P*	Adjusted Odds Ratios (95% CI)	*P*
Age group , y	15–24	373/537 (69)	Reference	Reference		
	25–44	2346/3334 (70)	1.0 (0.9–1.3)	.670		
	45–64	1253/1805 (69)	1.0 (0.8–1.2)	.985		
	≥65	322/537(60)	0.7 (0.5–0.8)	.001		
Sex	Male	1986/2837 (70)	Reference			
	Female	2306/3374 (68)	0.9 (0.8–1.0)	.159		
History of smoking	No	3438/5029 (68)	Reference			
	Yes	802/1107 (72)	1.2 (1.1–1.4)	.008		
History of alcohol	No	3284/4848 (68)	Reference			
	Yes	958/1293 (74)	1.4 (1.2–1.6)	<.001		
Site	Edendale	1442/2247 (64)	Reference			
	Tshepong	2857/3980 (72)	1.4 (1.3–1.6)	<.001	1.4 (1.2–1.7)	<.001
Cough	No	156/253 (62)	Reference			
	Yes	3696/5051 (73)	1.7 (1.3–2.2)	<.001	2.1 (1.5–2.8)	<.001
Night sweats	No	1481/2145 (69)	Reference			
	Yes	2644/3659 (72)	1.2 (1.0–1.3)	.009	1.3 (1.1–1.6)	.001
Influenza	Negative	4013/5823 (69)	Reference			
	Positive	239/325 (74)	1.3 (1.0–1.6)	.080		
Viral coinfection	No	2994/4342 (69)	Reference			
	Yes	1258/1806 (70)	1.0 (0.9–1.2)	.591		
HIV status	Negative	945/1407 (67)	Reference			
	Positive	3134/4445 (71)	1.2 (1.0–1.3)	.017		
Underlying medical condition other than HIV^a^	No	3931/5643 (70)	Reference			
	Yes	363/570 (64)	0.7 (0.6–0.8)	<.001		
Diabetes	No	3812/5432 (70)	Reference			
	Yes	120/212 (57)	0.5 (0.4–0.7)	<.001	0.5 (0.4–0.8)	.001
Worked in mine	No	2751/3609 (76)	Reference			
	Yes	351/416 (84)	1.7 (1.3–2.2)	<.001	1.6 (1.1–2.2)	.007
Pneumococcal coinfection^b^	No	3532/5174 (68)	Reference			
	Yes	456/605 (75)	1.4 (1.2–1.7)	<.001	1.3 (1.0–1.8)	.039
Duration of symptoms before admission, d	≤7	1586/2324 (68)	Reference			
	>7	2579/3673 (70)	1.1 (1.0–1.2)	.107		
Oxygen	No	2474/3625 (68)	Reference			
	Yes	1404/1944 (72)	1.2 (1.1–1.3)	.003		
On antibiotics	No	166/282 (59)	Reference			
	Yes	4077/5784 (70)	1.7 (1.3–2.1)	<.001		
ICU admission	No	4212/6094 (69)	Reference			
	Yes	18/32 (56)	0.6 (0.3–1.2)	.121		
Duration of hospitalization, d	<3	715/1087 (66)	Reference			
	3–7	1802/2503 (72)	1.3 (1.1–1.6)	<.001	1.1 (0.9–1.5)	.275
	>7	1551/2326 (67)	1.0 (0.9–1.2)	.603	0.8 (0.6–0.9)	.036
Died	No	3749/5286 (71)	Reference			
	Yes	433/777 (56)	0.5 (0.4–0.6)	<.001	0.5 (0.4–0.6)	<.001

Abbreviations: HIV, human immunodeficiency virus; ICU, intensive care unit, TB, tuberculosis.

^a^Underlying conditions included any of the following: asthma, other chronic lung disease, chronic heart disease (valvular heart disease, coronary artery disease, or heart failure excluding hypertension), liver disease (cirrhosis or liver failure), renal disease (nephrotic syndrome, chronic renal failure), immunocompromising conditions excluding HIV infection (organ transplant, immunosuppressive therapy, immunoglobulin deficiency, malignancy), neurological disease (cerebrovascular accident, spinal cord injury, seizures, neuromuscular conditions), or pregnancy. Comorbidities were considered absent in cases for which the medical records stated that the patient had no underlying medical condition or when there was no direct reference to that condition.

^b^Positive on *lyt*A polymerase chain reaction.

**Figure 1.**
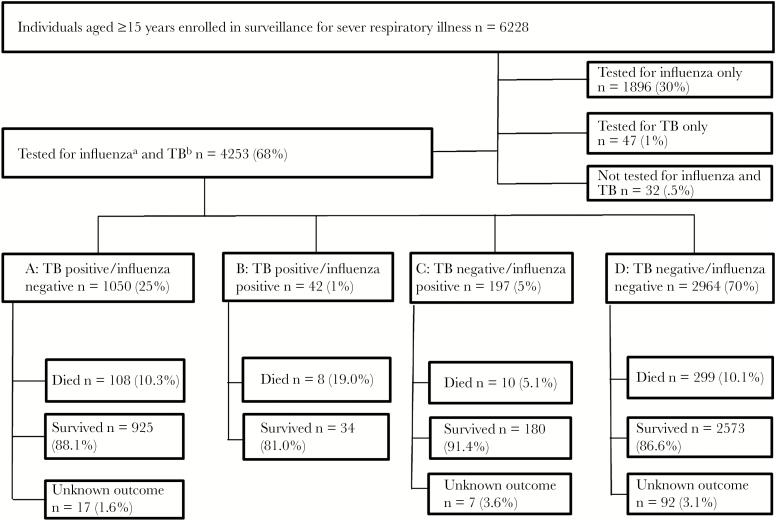
Individuals aged ≥15 enrolled in surveillance for severe respiratory illness, by tuberculosis and influenza status and in-hospital outcome, at Tshepong and Edendale Hospitals, South Africa, June 2010–May 2016. A, B, Tuberculosis (TB)-associated mortality analysis. B, C, Influenza-associated mortality. ^a^Two hundred thirty-nine (6%) positive for influenza. ^b^One thousand ninety-two (26%) positive for TB.

In total, 68% (4253/6228) of participants were tested for both pathogens (Figure 1). Among these individuals, the overall detection rate was 6% (239/4253) for influenza, 26% (1092/4253) for tuberculosis, and 77% (3113/4053) for HIV. Of these, 25% (1050/4253) were positive for tuberculosis only, 5% (197/4253) were positive for influenza only, and 1% (42/4253) were coinfected with influenza and tuberculosis. Of the 1092 individuals positive for tuberculosis, 444 (41%), 202 (19%), 114 (10%), and 332 (30%) were positive on PCR only, microscopy only, culture only, and on ≥2 tests, respectively. Of the 1087 individuals positive for tuberculosis with data on previous tuberculosis treatment, 118 (11%) had a history of being on tuberculosis treatment within 12 months of current admission.

### Factors Associated With Mortality in Patients Testing Tuberculosis Positive

Of the 1092 patients aged ≥15 years tested for influenza with laboratory-confirmed tuberculosis, 116/1075 (11%) died. Mortality was higher among individuals aged ≥65 years: 19% (7/36) compared with 6% (8/129) in the 15–24 age group (aOR, 3.6; 95% CI, 1.2–11.0) (Table 2). Patients with symptom duration longer than 7 days were more likely to die (12%, 94/786, vs 8%, 20/258; aOR, 1.5; 95% CI, 0.9–2.5), although this was not statistically significant on multivariable analysis. HIV-infected individuals were less likely to die (10%, 87/848, vs 12%, 20/168; aOR, 0.9; 95% CI, 0.5–1.6), although this was not statistically significant. On multivariable analysis controlling for HIV status and duration of symptoms, factors independently associated with death among tuberculosis-positive patients were age group ≥65 years compared with 15–24 years (aOR, 3.6; 95% CI, 1.2–11.0) and influenza coinfection (aOR, 2.3; 95% CI, 1.1–5.2). Coinfection with other respiratory viruses was not associated with increased mortality (unadjusted OR, 0.7; 95% CI, 0.4–1.1).

**Table 2.** Factors Associated With Death Among Hospitalized Individuals Aged ≥15 Years With Severe Respiratory Illness Testing Tuberculosis Positive (Tuberculosis Disease) at 2 Sentinel Surveillance Sites, Edendale Hospital and Tshepong Hospital, South Africa, 2010–2016 (n = 1075)

Characteristics		Case Fatality Ratio (%)	Univariate Analysis		Multivariable Analysis	
			OR (95% CI)	*P*	OR (95% CI)	*P*
Demographic characteristics			Reference			
Age group, y	15–24	8/125 (6)	Reference			
	25–44	69/656 (11)	1.7 (0.8–3.7)	.161	1.5 (0.7–3.3)	.286
	45–64	32/258 (12)	2.1 (1.0–4.6)	.077	1.8 (0.8–4.0)	.181
	≥65	7/36 (19)	3.5 (1.2–10.5)	.024	3.6 (1.2–11.0)	.026
Sex	Male	71/512 (14)	Reference			
	Female	45/563 (8)	0.5 (0.4–0.8)	.002		
Site	Edendale	39/301 (13)	Reference			
	Tshepong	77/774 (10)	0.7 (0.5–1.1)	.155		
Cough	No	3/32 (9)	Reference			
	Yes	104/960 (11)	1.2 (0.4–3.9)	.794		
Night sweats	No	39/306 (13)	Reference			
	No	74/746 (10)	0.8 (0.5–1.1)	.180		
HIV status	Negative	20/168 (12)	Reference			
	Positive	87/848 (10)	0.8 (0.5–1.4)	.526	0.9 (0.5–1.6)	.756
Underlying medical condition^a^	No	111/1032 (11)	Reference			
	Yes	5/43 (12)	1.1 (0.4–2.8)	.857		
Diabetes	No	113/1056 (11)	Reference			
	Yes	3/19 (16)	1.6 (0.5–5.5)	.482		
Smoking	No	92/858 (11)	Reference			
	Yes	24/202 (12)	1.1 (0.7–1.8)	.635		
Alcohol	No	95/831 (11)	Reference			
	Yes	21/229 (9)	0.8 (0.5–1.3)	.333		
Influenza infection	No	108/1033 (10)	Reference			
	Yes	8/42 (19)	2.0 (0.9–4.5)	.084	2.3 (1.0–5.2)	.045
Pneumococcal coinfection^b^	No	100/922 (11)	Reference			
	Yes	9/73 (12)	1.2 (0.6–2.4)	.696		
Viral coinfection^c^	No	92/773 (12)	Reference			
	Yes	22/269 (8)	0.7 (0.4–1.1)	.094		
Duration of symptoms before admission, d	≤7	20/258 (8)	Reference			
	>7	94/786 (12)	1.6 (1.0–2.7)	.062	1.5 (0.9–2.5)	.114
Duration of hospitalization, d	<3	20/189 (11)	Reference			
	3–7	52/480 (11)	1.0 (0.6–1.8)	.925		
	>7	42/371 (11)	1.1 (0.6–1.9)	.792		

Abbreviations: CI, confidence interval; OR, odds ratio.

^a^Asthma, other chronic lung disease, chronic heart disease (valvular heart disease, coronary artery disease, or heart failure excluding hypertension), liver disease (cirrhosis or liver failure), renal disease (nephrotic syndrome, chronic renal failure), immunocompromising conditions excluding HIV infection (organ transplant, immunosuppressive therapy, immunoglobulin deficiency, malignancy), neurological disease (cerebrovascular accident, spinal cord injury, seizures, neuromuscular conditions), or pregnancy. Comorbidities were considered absent in cases for which the medical records stated that the patient had no underlying medical condition or when there was no direct reference to that condition.

^b^Positive on *lytA* polymerase chain reaction.

^c^Coinfection with at least 1 of: parainfluenza virus 1, 2, and 3; respiratory syncytial virus; enterovirus; human metapneumovirus; adenovirus; rhinovirus.

### Factors Associated With Mortality in Patients Testing Influenza Positive

Among 239 hospitalized patients aged ≥15 years tested for tuberculosis with laboratory-confirmed influenza, 18/232 (8%) died. Mortality increased with increasing age: 6% (8/131; aOR, 1.0; 95% CI, 0.1–8.9), 10% (6/57; aOR, 2.5: 95% CI, 0.3–24.2), and 12% (3/25; aOR, 5.1; 95% CI, 0.4–65.9) among individuals aged 25–44, 45–64, and ≥65 years compared with the 15–24 age group (5%, 1/19). Mortality was higher in individuals with symptom duration >7 days (10%, 12/120) compared with symptoms ≤7 days (5%, 6/109; aOR, 1.6; 95% CI, 0.6–4.7), although these were not statistically significant on multivariable analysis (Table 3). HIV-infected individuals were more likely to die (9%, 14/156, vs 6%, 20/168; aOR, 2.0; 95% CI, 0.5–8.8), although this was not statistically significant.

**Table 3.** Factors Associated With Death Among Hospitalized Individuals Aged ≥15 Years With Severe Respiratory Illness and Testing Influenza Positive at 2 Sentinel Sites, Tshepong and Edendale Hospitals, South Africa, 2010–2016 (n = 232)

Characteristics		Case Fatality Ratio (%)	Univariate Analysis		Multivariable Analysis	
			OR (95% CI)	*P*	OR (95% CI)	*P*
Demographic characteristics						
Age group, y	15–24	1/19 (5)	Reference			
	25–44	8/131 (6)	1.2 (0.1–9.9)	.885	1.0 (0.1–8.9)	.989
	45–64	6/57 (11)	2.1 (0.2–18.8)	.501	2.5 (0.3–24.3)	.417
	≥65	3/25 (12)	2.5 (0.2–25.7)	.453	5.1 (0.4–65.9)	.210
Sex	Male	9/91 (10)	Reference			
	Female	9/141 (6)	0.6 (0.2–1.6)	.333		
Site	Edendale	3/70 (4)	Reference			
	Tshepong	15/162 (9)	2.3 (0.6–8.1)	.205		
Cough	No	0/4 (0)	Reference			
	Yes	15/188 (8)	1.0	<.001		
Night sweats	No	8/93 (9)	Reference			
	Yes	10/127 (8)	0.9 (0.3–2.4)	.846		
HIV status	Negative	4/66 (6)	Reference			
	Positive	14/156 (9)	1.5 (0.5–4.8)	.470	2.0 (0.5–8.8)	.337
Underlying medical condition other than HIV^a^	No	18/205 (9)	Reference			
	Yes	0/26 (0)	1.0			
Diabetes	No	17/223 (8)	Reference			
	Yes	1/7 (14)	2.0 (0.2–17.8)	.526		
Smoking	No	11/189 (6)	Reference			
	Yes	7/43 (16)	3.1 (1.1–8.7)	.027		
Alcohol	No	15/192 (9)	Reference			
	Yes	3/40 (8)	0.9 (0.3–3.5)	.946		
Worked in mine	No	8/129 (6)	Reference			
	Yes	2/9 (22)	4.3 (0.8–24.3)	.097		
Tuberculosis	No	10/190 (5)	Reference			
	Yes	8/42 (19)	4.2 (1.6–11.5)	.005	4.5 (1.5–13.3)	.007
Pneumococcal coinfection^b^	No	15/179 (8)	Reference			
	Yes	2/33 (6)	0.7 (0.2–3.2)	.654		
Viral coinfection^c^	No	14/194 (7)	Reference			
	Yes	4/36 (11)	1.6 (0.4–5.6)	.608		
Duration of symptoms before admission, d	≤7 d	6/109 (6)	Reference			
	>7	12/120 (10)	1.9 (0.7–5.3)	.213	1.6 (0.6–4.7)	.355
Duration of hospitalization, d	<3	4/54 (7)	Reference			
	3–7	9/96 (9)	1.3 (0.4–4.4)	.682		
	>7	4/76 (5)	0.7 (0.2–2.9)	.618		

Abbreviations: CI, confidence interval; OR, odds ratio.

^a^Asthma, other chronic lung disease, chronic heart disease (valvular heart disease, coronary artery disease, or heart failure excluding hypertension), liver disease (cirrhosis or liver failure), renal disease (nephrotic syndrome, chronic renal failure), immunocompromising conditions excluding HIV infection (organ transplant, immunosuppressive therapy, immunoglobulin deficiency, malignancy), neurological disease (cerebrovascular accident, spinal cord injury, seizures, neuromuscular conditions), or pregnancy. Comorbidities were considered absent in cases for which the medical records stated that the patient had no underlying medical condition or when there was no direct reference to that condition.

^b^Positive on *lytA* polymerase chain reaction.

^c^Coinfection with at least 1 of: parainfluenza virus 1, 2, and 3; respiratory syncytial virus; enterovirus; human metapneumovirus; adenovirus; rhinovirus.

On multivariable analysis controlling for age, HIV status, and symptom duration, laboratory-confirmed tuberculosis was associated with an increased risk of death in patients who tested positive for influenza (aOR, 4.5; 95% CI, 1.5–13.3) (Table 3). Coinfection with other respiratory viruses was not significantly associated with increased mortality (unadjusted OR1.6;95% CI,0.4-5.6).

## Discussion

Influenza and tuberculosis are both common etiologies identified among adults hospitalized with LRTI in South Africa, and both are associated with a high proportion of in-hospital mortality (12% and 11%, respectively). Although coinfection with influenza and tuberculosis was relatively rare (1%, of which 19% died [8/42]), tuberculosis coinfection was associated with elevated mortality risk (aOR, 4.5) among patients with influenza, and influenza coinfection was associated with increased mortality (aOR, 2.3) among patients with tuberculosis. These results suggest that when influenza and tuberculosis disease coexist in an individual patient, their copresence is associated with a worse prognosis than either infection alone.

In our study, we found an increased risk of mortality among tuberculosis-positive patients who were coinfected with influenza viruses. Ecological studies have suggested excess tuberculosis mortality during influenza pandemics and seasonal epidemics; however, a causal relationship could not be demonstrated [6, 26–29]. Our results are similar to reports from descriptive studies from tuberculosis sanatoria in the 1950s, which also reported increased tuberculosis mortality or increased complications following influenza infection [30, 31]. However, these were not analytical studies. Animal experimental studies have suggested a causal relationship between influenza infection and tuberculosis mortality, suggesting that influenza infection of mice with tuberculosis reduced long-term survival [32–34]. Bernard et al. showed that in mice, an influenza challenge 1–5 weeks after tuberculosis infection, compared with tuberculosis only–infected mice, resulted in 50%–75% shorter survival time and a higher case fatality ratio [33]. In the same study, sequential infection of mice with tuberculosis and influenza, with tuberculosis infection on day 0 and influenza infection 7 weeks later, resulted in increased pulmonary disease and a delay in mycobacterium clearance from the lungs of infected mice [34].

In our study, we also found an increased risk of mortality among influenza-positive patients who were coinfected with tuberculosis. Our results are similar to those from studies conducted during the 2009 influenza pandemic, which either reported a high prevalence of tuberculosis in individuals who were hospitalized or died of influenza compared with the general population or described tuberculosis as a risk factor for influenza-associated severe disease [8–10]. However, these studies were mostly descriptive. In animal studies, Redford et al. demonstrated that influenza A virus infection of mice 28 days before or during *M. tuberculosis* infection impaired mycobacterium control and decreased host survival [35]. Coinfection and superinfection with other bacterial pathogens, *Streptococcus pneumoniae, Staphylococcus aureus,* and *Haemophilus influenzae,* have also been reported as risk factors for severe influenza-associated disease and death [36, 37].

We had previously reported an increased risk of death among patients with tuberculosis who were coinfected with influenza, which was only significant among patients with symptoms >7 days [20]. In the current study, we used a different analytical approach to explore the effects of tuberculosis and influenza separately in the groups of individuals with each pathogen infection. Using this approach, although not statistically significant, patients with longer duration of symptoms compared with those with more acute presentation were at increased risk of death. It is possible that the association with severe outcomes in tuberculosis-positive patients, who are coinfected with influenza, is in part due to the chronic lung damage caused by tuberculosis. In an experimental study, Bernard et al. demonstrated that the amount of tissue damage among tuberculosis–influenza-coinfected mice increased with increasing time of tuberculosis infection before the influenza challenge [33]. Studies restricted to influenza patients with an acute presentation did not find an association between coinfection and severe outcomes, although in 1 such study, tuberculosis was associated with severe disease on univariate analysis [38, 39]. A study from Thailand did not find an increased risk of clinical worsening or mortality among hospitalized tuberculosis patients coinfected with influenza presenting with acute respiratory illness [39]. In a case series of patients with concurrent influenza and tuberculosis in South Korea, there were no deaths among the coinfected [40]. This may suggest that chronic lung damage secondary to tuberculosis plays an important role in disease presentation and outcome. However, in our analysis, we did not have data on radiological changes in the lungs to assess whether chronic lung damage was associated with severe outcomes.

Our study has limitations that warrant discussion. First, whereas we obtained a tuberculosis result in 69% of patients admitted with SRI, due to logistic limitations including contraindications to induced sputum collection, we were not able to offer all patients sputum induction, and not all patients provided an expectorated sputum specimen. Other patients were not able to produce a sputum specimen even after sputum induction procedures. We may have missed patients with more severe illness who died before tuberculosis testing or were too ill to expectorate as patients who were tested compared with those not tested were less likely to die during current admission. This could have led to underestimation of case fatality ratios. Second, microbiologic confirmation of a diagnosis of tuberculosis is challenging, especially in patients who are infected with HIV [41, 42]. HIV-infected individuals made up 77% of participants in our study; therefore, we may have misclassified some cases as not having tuberculosis following a negative microbiologic result, leading to potential underestimation of the association with severe outcomes in patients who are coinfected. HIV infection is a risk for death for both influenza and tuberculosis and could be an effect modifier in the association between coinfection and mortality. However, we were not powered to look at HIV-infected and HIV-uninfected separately. Third, we did not have sufficient data to adjust for the different levels of severity at the time of admission. Due to the small numbers of patients who died, we were not able to use propensity score for our analyses.

In conclusion, we observed an increased risk of mortality in patients coinfected with influenza and tuberculosis, compared with individuals with either tuberculosis or influenza alone. This study, using laboratory-confirmed, individual-level data, supports the hypothesis from ecological studies suggesting that influenza–tuberculosis coinfection is associated with increased mortality. These data may be useful to guide clinicians and policy makers, especially in countries with high burden of tuberculosis and HIV and limited resources, in making decisions on whether influenza vaccines or influenza antivirals should be prioritized for tuberculosis patients and whether investigating for tuberculosis in patients with influenza should be considered.
